# Supplementary material for: Neurohormonal signaling via a sulfotransferase antagonizes insulin-like signaling to regulate a Caenorhabditis elegans stress response
Source: Nat Commun. 2018 Dec 4;9:5152. doi: 10.1038/s41467-018-07640-w (PMC6279808; doi:10.1038/s41467-018-07640-w)

Supplementary Discussion

We attempted to rescue the loss of *sod-5::GFP* expression in *nhr-1* mutants by expressing a wild-type copy of *nhr-1* under the control of a 3 kb fragment of its endogenous promoter but were unable to recover any transgenic animals. We suspect that overexpression of *nhr-1* in one or more tissues in embryos promoted developmental arrest and hence prevented recovery of transgenic animals. Alternatively, it is possible that the 3 kb fragment used does not include the entire *nhr-1* promoter (the next closest gene to *nhr-1* is >25 kb away) and that *nhr-1* was not expressed in the right place or at the right time to rescue the loss of *nhr-1*. In addition, there are three possible isoforms of *nhr-1*, and it is possible that the isoform we expressed (R09G11.2c) is not the isoform that is functional in embryos to control this particular response to osmotic stress.

We also attempted to test if tissue-specific expression of a wild-type copy of *nhr-1* could rescue the loss of *sod-5::GFP* expression in *nhr-1* mutants, we expressed a wild-type copy of *nhr-1* fused to mCherry in the intestine (using the *ges-1* promoter), neurons (using the *rab-3* promoter), or muscle (using the *unc-54* promoter). We were unable to detect *nhr-1::mCherry* expression in these transgenic embryos and tissue-specific expression of *nhr-1* did not restore *sod-5::GFP* expression in response to 500 mM NaCl in embryos. We suspect that these transgenic animals did not express NHR-1 sufficiently early in embryonic development to promote *sod-5::GFP* expression in embryos.

Supplementary Figure 1 SSU-1 is not required for DAF-16 translocation into the nucleus in response to osmotic stress**.** Confocal images of DAF-16::GFP localization after 5 hrs of exposure to 500 mM NaCl in wild-type and *ssu-1(fc73)* mutant embryos. Scale bars, 10 µm. The red dashed lines indicate embryos scored as predominantly nuclear DAF-16 localization. The white dashed lines indicate embryos scored as predominantly cytoplasmic DAF-16 localization. Images with a red border were used as representative images in Fig. 3f.

WT 50mM salt
(1/14 predominantly nuclear)

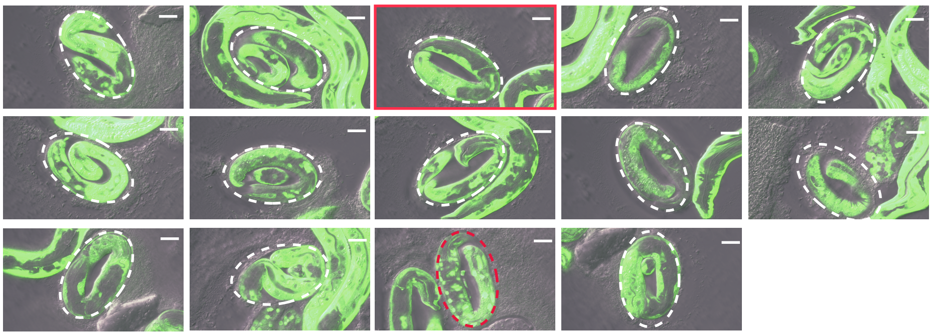

WT 500mM salt
(11/14 predominantly nuclear)

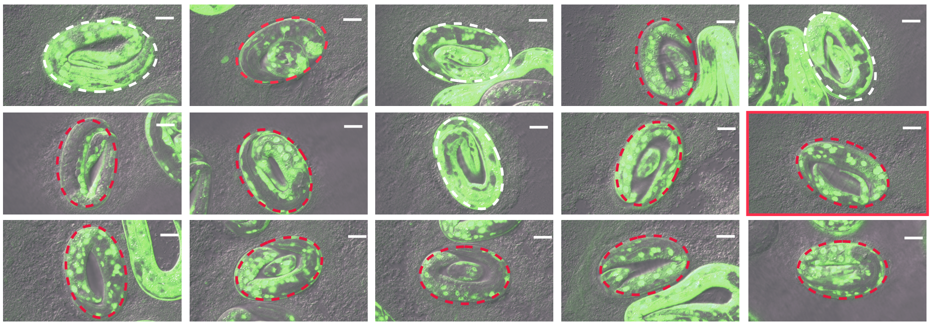

***ssu-1(-)* 50mM salt**
(1/15 predomintantly nuclear)

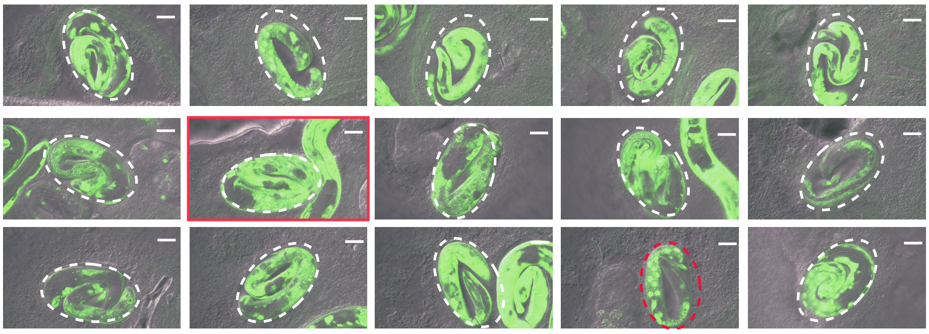

***ssu-1(-)* 500mM salt**
(9/14 predominantly nuclear)

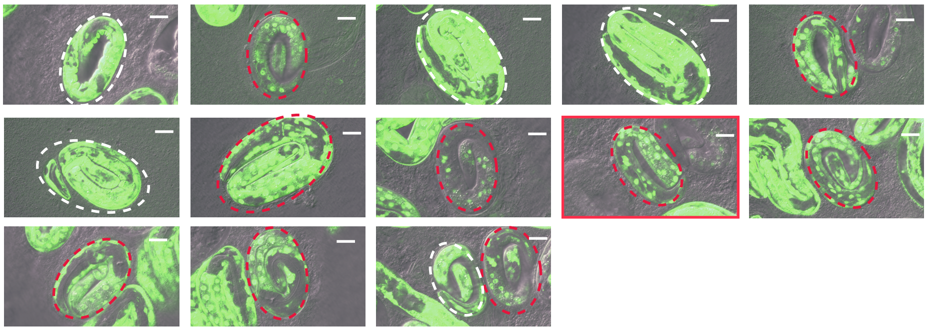

Supplement: Supplementary file 1 — Supplementary Information [file 41467_2018_7640_MOESM1_ESM.pdf]
