# Supplementary material for: Neurohormonal signaling via a sulfotransferase antagonizes insulin-like signaling to regulate a Caenorhabditis elegans stress response
Source: Nat Commun. 2018 Dec 4;9:5152. doi: 10.1038/s41467-018-07640-w (PMC6279808; doi:10.1038/s41467-018-07640-w)
Supplement: Supplementary file 5 — Description of Additional Supplementary Files [file 41467_2018_7640_MOESM5_ESM.pdf]

Description of Additional Supplementary Files

Supplementary Dataset 1: Profile of mRNA expression in wild-type and ssu-1(fc73) embryos at 50 mM NaCl and 500 mM NaCl.

Supplementary Dataset 2: Profile of mRNA expression in wild-type and daf-16(m26) embryos at 50 mM NaCl and 500 mM NaCl.

Supplementary Dataset 3: Profile of lipid and polar metabolites in wild-type, ssu-1(fc73), and daf-16(m26) embryos from parents grown at 50 mM and 300 mM NaCl.
